# Osteogenic Potential of Simvastatin and Fluvastatin in an Organotypic Bone Model

**DOI:** 10.3390/ph18070939

**Published:** 2025-06-21

**Authors:** Lukas Poskevicius, Victor Martin, Guilherme Costa, Gintaras Juodžbalys, Pedro Sousa Gomes

**Affiliations:** 1 Faculty of Odontology, Lithuanian University of Health Sciences, 44307 Kaunas, Lithuania; 2 BoneLab, Faculdade de Medicina Dentária, Universidade do Porto, Rua Dr. Manuel Pereira da Silva, 4200-393 Porto, Portugal; vmartin@fmd.up.pt (V.M.);; 3 LAQV/REQUIMTE, Faculdade de Medicina Dentária, Universidade do Porto, Rua Dr. Manuel Pereira da Silva, 4200-393 Porto, Portugal

**Keywords:** bone, fluvastatin, osteogenesis, simvastatin, organotypic model

## Abstract

**Background/Objectives**: Statins, widely prescribed for their lipid-lowering properties, also exert pleiotropic effects on various tissues, including bone. However, their osteogenic potential remains poorly defined due to variability in statin type, dosage, and experimental models. This study investigates the osteogenic effects of fluvastatin (FV) and simvastatin (SV) on the ex vivo embryonic chick femur model. **Methods**: Femora were cultured with logarithmic concentrations (0.1–10 µM) of FV or SV, followed by characterization via microcomputed tomography, histological analysis, and quantitative gene expression. **Results**: Both statins enhanced osteogenic outcomes at low concentrations (0.1–1 µM), as evidenced by increased bone volume fraction, trabecular organization, collagen matrix maturation, and mineral deposition. Molecular analysis revealed upregulation of key osteogenic markers—RUNX2, SPP1, and COL1A2—with no significant change in chondrogenic markers (SOX9, ACAN), indicating selective activation of osteogenic pathways. In contrast, higher-dose treatment (10 µM) attenuated these effects. **Conclusions**: These findings underscore the dose-dependent osteoinductive potential of statins and support their application in bone repair strategies within carefully defined therapeutic windows.

## 1. Introduction

Statins are a class of widely prescribed lipid-lowering agents that inhibit cholesterol biosynthesis by blocking 3-hydroxy-3-methylglutaryl coenzyme A (HMG-CoA) reductase, a key rate-limiting enzyme in the mevalonate pathway [1]. Originally developed to manage hypercholesterolemia, statins have proven effective in reducing the incidence of cardiovascular events associated with hyperlipidemia and atherosclerosis [2]. These compounds can be categorized based on their origin (natural or synthetic), generation, and lipophilicity—factors that influence their potency and pharmacokinetic properties [3]. Statins are among the most widely prescribed medications worldwide, with approximately 35% of the adult population in the United States reported to be under statin therapy [4,5].

Beyond their lipid-lowering capacity, statins are also recognized for a range of pleiotropic effects that extend to distinct tissues and organ systems. These include pro-angiogenic, antioxidant, anti-inflammatory, and immunomodulatory activities, as well as a positive impact on bone tissue [6,7,8,9]. Despite the promising data, the outcomes of statin-based interventions on bone remain inconsistent across studies. This variability likely stems from differences in administration routes, statins type, dosage, and study design [10]. While systemic administration often yields modest or variable effects on bone metabolism [11,12,13], local application of statins has shown more consistent anabolic outcomes [14,15]. Despite this realization, the optimal dosing strategy for achieving pro-osteogenic effects without eliciting cytotoxic responses remains unresolved. Furthermore, although lipophilic statins are generally considered more favorable for bone applications due to their higher cell membrane permeability, most studies have focused narrowly on simvastatin, often without exploring comparative efficacy across a broader pharmacological range [1].

In vitro experimental models are crucial to evaluate molecular and cellular effects of statins in bone cells; however, they fail to replicate the complexity of native tissue architecture, including 3D structure, cell–cell communication, and extracellular matrix (ECM) organization [16]. Cells in these simplified systems often lack the contextual signals provided by their native microenvironment, which significantly modulate gene expression and differentiation [17]. In contrast, in vivo models offer physiological relevance, but are limited by biological variability, confounding systemic influences, and ethical constraints due to high animal usage [18,19,20].

To bridge this gap, ex vivo models have been regarded as valuable research systems for bone-related applications, combining the structural integrity of native tissue with experimental accessibility and control [21,22]. One such model is the organotypic culture of embryonic chicken femora, which preserves osteo-chondrogenic cell populations and native tissue architecture. This model enables the investigation of bone development and remodeling under defined experimental conditions, offering a platform that is both ethically advantageous and translationally relevant due to the conservation of key ossification pathways between birds and mammals [23].

Hence, this study aims to investigate the osteogenic effects of two statins—fluvastatin (a first-generation synthetic statin) and simvastatin (a second-generation semi-synthetic statin)—across a wide, logarithmic concentration range using the ex vivo embryonic chick femur model.

Statin dosages reported in the literature vary widely depending on the therapeutic application, with systemic and local delivery approaches yielding markedly different exposure levels [24]. The logarithmic concentration range chosen in this study was designed to encompass this variability, providing a basis for the identification of statin concentrations most conducive to bone-related therapeutic applications in future investigations. To the best of the authors’ knowledge, this is the first study to assess statin effects using an organotypic femoral model, encompassing an integrative evaluation of cellular organization, tissue growth and structure, matrix mineralization, and osteogenesis-associated gene expression.

## 2. Results

### 2.1. Microtomographic Analysis

**Figure 1:**
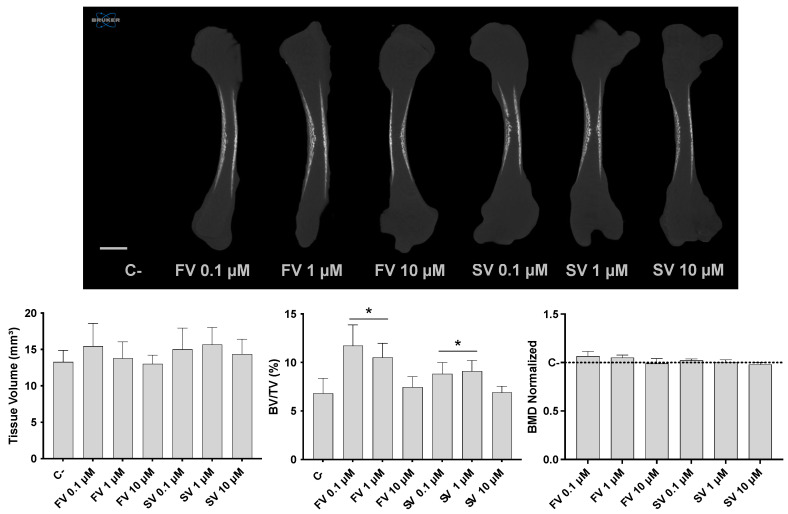
Three-dimensional microtomographic analysis. Upper panel: representative coronal sections of the femora reconstructions. Lower panel: quantitative analysis of tissue volume (TV), bone volume fraction (BV/TV), and bone mineral density (BMD). * *p* ≤ 0.05 vs. control.

Micro-CT analysis revealed distinct effects in bone morphometric parameters following statin treatment (Figure 1). Tissue volume (TV) remained relatively consistent across all experimental groups, indicating that overall femur size was not significantly influenced by the treatments. On the other hand, the bone volume fraction (BV/TV) was significantly increased in femora treated with FV and SV at 0.1 and 1 μM, indicating an elevated proportion of mineralized bone relative to the total tissue volume. Notably, bone mineral density (BMD) remained comparable across all groups, with no significant differences relative to the control.

### 2.2. Histological Evaluation

**Figure 2:**
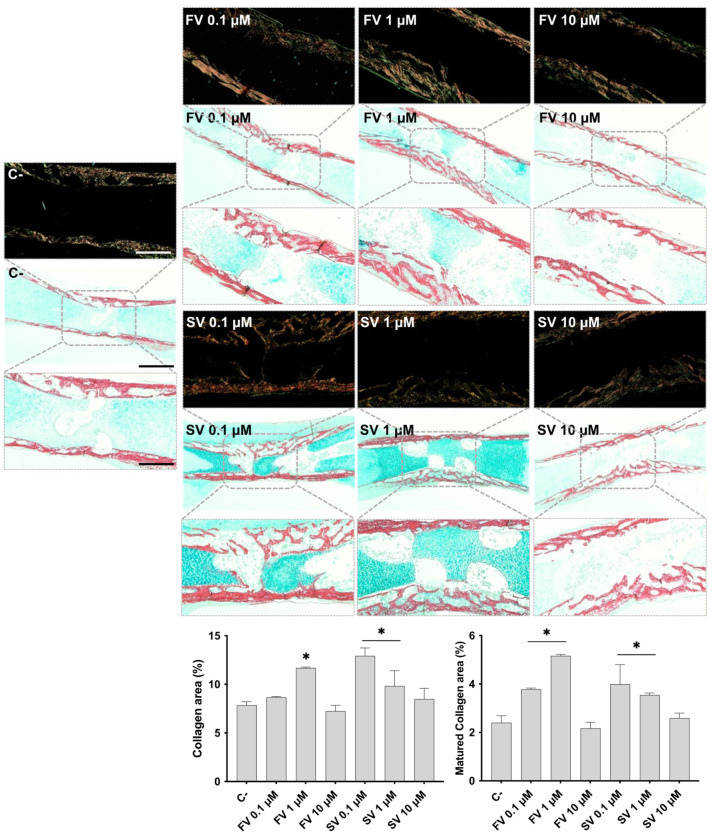
Representative images of Alcian Blue and Sirius Red (AB/SR) staining under brightfield (light background) and polarized light (dark background). The graphics refer to the quantitative analysis of the SR-stained area (collagen area) and mature collagen area. * *p* ≤ 0.05 vs. control. Scale bar corresponds to 100 µm or 200 µm upon selected insets.

Femora cultivated in all conditions were analyzed by histochemical staining, with a particular emphasis on the diaphyseal region. Figure 2 shows representative tissue sections stained with Alcian blue (AS), highlighting the proteoglycan-rich cartilage matrix, and Sirius red (SR), marking the collagenous matrix distribution. In control samples, the development of a collagenous matrix from the periosteal region progressing centripetally into to the inner region is noteworthy, extending from the mid-diaphyseal region into the epiphysis, as revealed by SR staining, forming a trabecular-like arrangement. Meanwhile, the bluish staining of the proteoglycan-rich cartilage matrix can still be seen in the innermost section of the diaphysis. Femora exposed to FV, particularly at lower concentrations (0.1 μM and 1 μM), exhibited a more mature and developed collagenous matrix within the diaphyseal region. At 0.1 μM, the collagen matrix appeared slightly thicker, with enhanced trabecular organization. This effect was maintained at 1 μM, with increased matrix deposition and a well-structured trabecular pattern. At 10 μM, a similar trend was observed, although the degree of trabecular organization appeared less pronounced compared to the lower concentrations. Femora treated with SV also exhibited features indicative of a more mature and developed collagenous matrix, particularly at lower concentrations. At 0.1 μM, samples showed a marked increase in SR-stained areas, accompanied by an expanded central region of the diaphysis with evident cell migration and trabecular organization. This trend was also evident at 1 μM, where increased matrix deposition and trabecular structuring were observed, although to a slightly lesser extent than at 0.1 μM. In contrast, femora exposed to 10 μM SV displayed a collagenous matrix organization comparable to that of the control, with limited evidence of further trabecular development. The histological appearance of the femora is consistent with the quantitative histochemical analysis of the collagenous area. The increased intensity and distribution of SR staining observed in samples treated with lower concentrations of both FV and SV align with the quantitative data, which indicate an expansion of the collagen-rich matrix.

Polarized light microscopy was employed to assess the birefringence properties of SR-stained sections, allowing for the evaluation of collagen fibril maturation based on color intensity and distribution. In femora treated with FV and SV, particularly at 0.1 μM and 1 μM, an increase in birefringent red-orange fibers was observed in the diaphyseal region, indicating enhanced collagen fibril maturation. These features were less pronounced at the highest concentration (10 μM), where the birefringence pattern more closely resembled that of the control samples. These qualitative observations were corroborated by quantitative histochemical analysis, which confirmed an increased area of mature collagen fibrils in the groups treated with low concentrations of fluvastatin and simvastatin.

**Figure 3:**
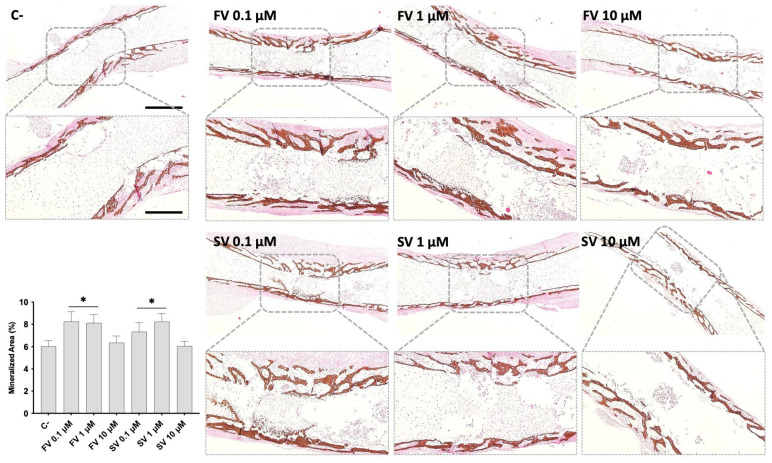
Representative images of Von Kossa (VK) staining of mineralized matrix. The graph refers to the quantitative analysis of the stained area. * *p* ≤ 0.05 vs. control. FV 10 µM and SV 10 µM. Scale bar corresponds to 100 µm or 200 µm upon selected insets.

Von Kossa staining was performed to evaluate mineral deposition within the diaphyseal region of cultured femora under different treatment conditions (Figure 3). In control femora, mineralized areas were primarily localized in the mid-diaphyseal region, developing centripetally. The mineral deposits appeared as discrete dark-brown regions along the bone surface. Femora treated with FV or SV at 0.1 μM and 1 μM showed a marked increase in VK-positive staining, indicating enhanced mineral deposition, and a more mature trabecular organization. Femora exposed to 10 μM FV or SV displayed mineralization patterns comparable to the control group, with less extensive staining and trabecular development. Quantitative histochemical analysis of VK-positive areas supports these observations.

### 2.3. Gene Expression Analysis

**Figure 4:**
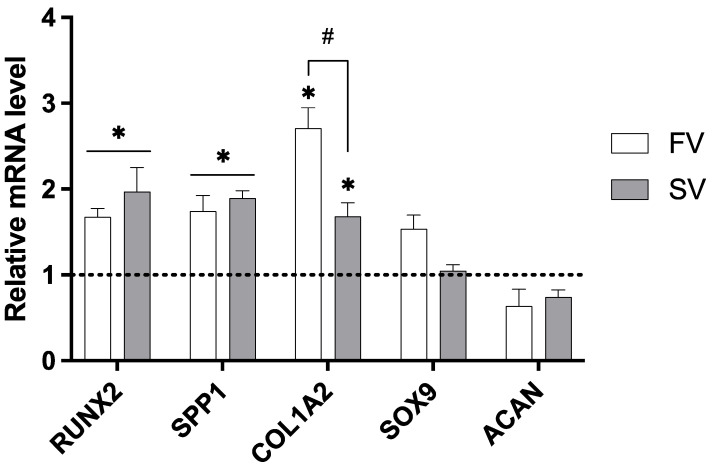
Relative gene expression of osteogenic and chondrogenic markers in cultures treated with 1 µM simvastatin or fluvastatin. Expression levels normalized to GAPDH. * *p* ≤ 0.05 vs. control; # *p* ≤ 0.05 vs. the other statin-treated group.

The expression of key genes associated with bone development was assessed in femora grown in media containing SV and FV at a concentration of 1 μM (Figure 4). When compared to the control group, SV significantly upregulated the expression of all osteogenic markers analyzed, including RUNX2, SPP1, and COL1A2, indicating a broad activation of osteogenic pathways. FV also significantly increased RUNX2 and SPP1 expression and showed an even greater upregulation of COL1A2 compared to simvastatin. Importantly, neither statin significantly affected the expression of the chondrogenic markers SOX9 and ACAN.

## 3. Discussion

The present study explored the dose-dependent effects of two statins—simvastatin and fluvastatin—on bone tissue dynamics, within an organotypic bone model. Statins, beyond their ability to reduce cholesterol biosynthesis and lipid-lowering clinical effectiveness, have been increasingly recognized for their pleiotropic effects on bone, particularly their potential to modulate osteogenic activity [25]. However, the therapeutic window and optimal dosing parameters for statins in bone-related applications remain poorly defined. Moreover, the mechanisms driving their osteogenic effects are not yet fully understood—particularly within experimental systems that preserve the native structural and functional complexity of developing bone tissue. To address these gaps, the present study utilizes a physiologically relevant ex vivo embryonic femur model that retains the 3D architecture, cell–cell interactions, and extracellular matrix organization characteristic of native bone. By integrating high-resolution microtomography, detailed histological analysis, and quantitative gene expression profiling, this multifaceted approach enables a comprehensive assessment of statin-induced osteogenesis. Importantly, our findings not only provide novel insights into the dose-dependent effects of fluvastatin and simvastatin but also validate key in vitro observations within a more translationally relevant ex vivo context.

Microtomographic data revealed a trend for an increased tissue volume (TV) in groups treated with low concentrations of SV and FV, suggesting a trend for increased cellular proliferation. In vitro studies have reported a dose-dependent effect of statins on the cell proliferation of osteoblastic precursor cells—i.e., mesenchymal stromal cells (MSCs): low concentrations within the nanomolar range generally promote proliferation [26], whereas higher concentrations tend to exert cytotoxic effects and reduce cell viability [27,28]. Although the precise threshold varies across different statins, concentrations exceeding 1 µM are broadly associated with decreased proliferation [29].

At lower concentrations, statins have been shown to exert cytoprotective and proliferative effects across various experimental models. In vitro and most in vivo studies report an upregulation of Bcl-2 expression following low-dose statin treatment, supporting a pro-survival role [30,31,32]. Within the bone microenvironment, Bcl-2 overexpression has been associated with enhanced osteoblastic proliferation [33], consistent with the increased tissue volume observed in the organotypic model of the present study. Moreover, low-dose statins have been found to promote MSCs proliferation, likely via the activation of the Wnt/β-catenin signaling pathway [26]. These proliferative effects have also been observed in cells growing in the presence of distinct biomaterials [34,35,36] and under distinct pathological conditions [37,38,39]. Further supporting this response, in vivo data demonstrates increased osteoblastic cellularity following SV administration [40,41].

Besides promoting cellular proliferation, low concentrations of statins also enhanced osteogenic differentiation and mineral deposition within the organotypic model. Furthermore, histochemical analysis of the collagenous matrix revealed a more mature trabecular architecture in the mid-diaphysis, as well as a significant increase in the collagen-rich area, both features indicating an advanced stage of osteogenic commitment. These histological findings were supported at the molecular level by the upregulation of key osteogenic markers. Notably, treatment with 1 µM of either simvastatin or fluvastatin led to increased expression of *RUNX2*, *SPP1*, and *COL1A2*, genes associated with early osteogenic transcriptional activation, matrix maturation, and collagen synthesis, respectively. Importantly, this osteoinductive response occurred without concurrent activation of cartilage-specific pathways. The expression levels of *SOX9* and *ACAN*—which encode a master transcription factor for chondrogenic lineage commitment and a major proteoglycan component of the cartilage extracellular matrix, respectively [42]—remained unchanged. This supports the notion that statins selectively promote osteogenic differentiation in this model, without modulating chondrogenic gene expression.

As part of the observed osteogenic enhancement, RUNX2 emerges as a central regulatory factor, essential for osteogenic commitment and functioning as a master transcriptional controller of osteoblast differentiation [43]. It orchestrates the expression of osteoblast-specific genes by activating multiple signaling pathways involved in bone formation [42]. Among its downstream targets are critical osteogenic markers such as osteocalcin (*OC*), osteopontin (*SPP1*), and collagen type I (*COL1*), all of which contribute to matrix maturation and mineralization [42]. Numerous in vitro studies have demonstrated that statins promote osteogenic differentiation by upregulating both the expression and activity of RUNX2, along with its downstream targets in MSCs [44,45]. Notably, RUNX2 itself is modulated by upstreaming signaling molecules, particularly bone morphogenetic proteins (BMPs), such as BMP-2—a pathway that statins have also been shown to activate [46].

Among the downstream targets of *RUNX2*, several genes are critically involved in the development and maturation of the bone extracellular matrix. Notably, *SPP1* and *COL1A2* contribute significantly to the structural and functional integrity of the osteogenic matrix. *SPP1,* coding for osteopontin, plays a relevant role in regulating mineral deposition and mediating cell–matrix adhesion. Its upregulation in the present study is consistent with previous in vivo findings, where local statin administration was associated with elevated osteopontin expression [47].

Similarly, *COL1A2*, which encodes the alpha-2 chain of type I collagen, plays a central role in providing the organic scaffold necessary for matrix assembly and subsequent mineralization. In line with the upregulation of *COL1A2*, collagenous matrix was found to be increased, particularly at the mid-diaphysis region, further evidencing a more mature fibril organization, particularly with 0.1 μM and 1 μM of fluvastatin and simvastatin. Previous in vitro studies with human osteoblastic populations reported an increased expression of collagen type I [48], an effect found to begin at early culture time points and be kept throughout the culture period, suggesting an increased accumulation of extracellular matrix in a time-dependent manner [6]. This evidence is further gathered from in vivo studies, in which SV administration was found to induce the formation of thicker collagen bundles at the peri-implantar space, upon implant placement in dogs [49]. Evidence from a bone-healing model further supported the enhanced activity of statins to promote enhanced collagen maturation, similarly attaining the data verified in the present report with an increased collagen mature area being attained with low concentrations of fluvastatin and simvastatin.

In addition to promoting osteoblastic differentiation, statins also appeared to support terminal osteogenic maturation, leading to enhanced mineral deposition. At low concentrations (0.1 and 1 μM), both simvastatin and fluvastatin significantly increased the formation of mineralized tissue, as evidenced by a significantly higher bone volume fraction (BV/TV) in microtomographic analysis. These findings were further corroborated by histomorphometric evaluation, which revealed a larger area of mineralized matrix in VK-stained sections at the same concentrations. Notably, these changes occurred without significant alterations in bone mineral density (BMD), suggesting that statins may stimulate the expansion of mineralizing surfaces or increase the number of matrix-producing osteoblasts, rather than altering the degree of mineralization within the existing matrix. This pattern is indicative of enhanced matrix production and progressive maturation, rather than accelerated mineral crystallization, aligning with early-stage bone tissue formation [50]. These observations are consistent with in vitro studies highlighting the ability of statins to induce matrix mineralization in osteoprogenitor populations, in parallel with the activation of the osteogenic transcriptional program [27,51], possibly via the activation of the Wnt/β-catenin signaling pathway [26]. Supporting this, in vivo studies related to implant placement have reported increased early-stage mineralized bone formation following statin administration [52,53], further validating the pro-mineralizing effects observed in the present organotypic model.

## 4. Material and Methods

### 4.1. Organotypic Culture of Embryonic Chick Femora with Statins

Fertilized chick eggs (*Gallus domesticus*) were incubated in an Octagon 40 ECO rotating egg incubator (Brinsea, Weston-Super-Mare, UK), at 37 °C and 50% humidity. On embryonic day 11 (E11), the fertilized eggs were opened, and the femora were dissected upon soft tissue removal. Then, isolated femora were transferred into culture inserts (Netwell Insert, Costar 3480; 440 μm pore diameter, Tewksbury, MA, USA) and placed into six-well plates (two femora per well), each containing 1 mL of culture medium. The culture medium consisted of alpha-Minimal Essential Medium (α-MEM; Gibco, Dublin, Ireland), supplemented with 1% (*v*/*v*) penicillin/streptomycin (Gibco, Waltham, MA, USA, 15240–062) and 1% (*v*/*v*) 2-phospho-L-ascorbic acid (Sigma-Aldrich, St. Luis, MO, USA, 49752-10G). Femora were incubated at 37 °C in a humidified atmosphere of 5% CO_2_ and placed at the air/liquid interface.

Femora were cultured either in control conditions or in the presence of statins. Fluvastatin (FV; batch 80034000) and simvastatin (SV; batch 80030510) were kindly provided by Bluepharma, Coimbra, Portugal. Stock solutions were prepared in dimethyl sulfoxide (DMSO) at a concentration of 70 mM. After an initial 6-day culture period in basal culture medium, treatment was initiated by replacing the medium with a fresh medium containing either FV or SV, at final concentrations of 0.1 μM, 1 μM, or 10 μM. Control cultures received an equivalent concentration of the highest amount of DMSO without statins. All media were replaced daily for the remaining 5 days of culture. For the subsequent analysis, femora samples were fixed with 4% paraformaldehyde for microtomographic or histological analysis, or frozen for gene expression analysis.

### 4.2. Microtomographic Analysis

High-resolution micro-computed tomography (micro-CT) was performed using a Skyscan 1276 System (Bruker, Kontich, Belgium, version 1.0.11). Samples were scanned at 40 kV, 100 μA, with an exposure time of 800 ms and a voxel size of 4.5 µm. The achieved projection images were three-dimensionally reconstructed, employing the NRecon Software (Bruker, version 1.7.4.2) pre-setting the fine-tuning configuration (ring reduction—4 and beam hardening—31%) and the grayscale range normalized to 0–0.25. The morphometric assessment of the bone was performed using CTAnalyser software (Bruker, version 1.17.7.2). The parameters calculated included tissue volume (TV), bone volume (BV), bone volume fraction (BV/TV), and bone mineral density (BMD). Representative 3D reconstructions were generated using CTVox software (Bruker, version 3.3.0).

### 4.3. Histological Preparation and Histochemical Staining

Following fixation, tissue samples were paraffin-embedded and sectioned. Sections were deparaffinized, and then rehydrated through a graded ethanol series followed by distilled water. Sections were stained in Weigert’s hematoxylin. After a rinse in tap water, the samples were stained in Alcian Blue (AB) solution, pH 2.5 (1 g Alcian Blue 8GX (Sigma-Aldrich, St. Luis, MO, USA, A-5268, CI 74240), 3 mL glacial acetic acid (Fisher), and 97 mL distilled water). The samples were rinsed in tap water and stained with a Sirius Red (SR) solution (0.1 g Sirius red F3B, direct red, CI 35780, Sigma Aldrich 36554-8) and 100 mL saturated aqueous picric acid, CI 10305 (Sigma Aldrich 925-40). After a rinse in 0.01 N HCl, samples were dehydrated and mounted [54]. Sections were analyzed under brightfield observation and polarized light microscopy to assess collagen birefringence, which distinguishes collagen fibril maturity, as more mature fibrils exhibit red birefringence, while less mature fibrils appear green [19]. To evaluate tissue mineralization, histological sections underwent Von Kossa (VK) staining. Sections were incubated in a 1% silver nitrate under ultraviolet light for 20 min. Subsequently, they were immersed in 5% sodium thiosulfate for 5 min to remove unreacted silver.

All histological samples were then examined using a Zeiss Axiolab 5 microscope and Axiocam 208 color image system (Zeiss, Oberkochen, Germany). Quantitative histomorphometric analyses (e.g., percentage of the collagen area, mature collagen area, and mineralized area) were performed upon segmentation applying the Otsu algorithm, using ImageJ software (version 1.53c). Assays were conducted in quintuplicates.

### 4.4. Gene Expression Analysis

Frozen femora were lysed in TRIzol ^®^ (Invitrogen, San Diego, CA, USA) and total RNA was extracted using chloroform, accordingly to the established manufacturer’s protocol. RNA concentration and purity (A260/280 ratio) were determined using a Take3 module on a Synergy HT microplate reader (BioTek, Winooski, VT, USA). The conversion to cDNA was conducted with a two-step reverse transcription quantitative NZY Kit (NZYTECH), normalizing the RNA concentration to 800 ng/µL, followed by RNAse (NZY) incubation. Quantitative PCR was performed on a CFX384 Real-Time PCR Detection System (Bio-Rad, Hercules, CA, USA) using iTaq Universal SYBR^®^ Green Supermix and optimized primers, displayed in Table 1 (all from Bio-Rad), following the Bio-Rad cycling protocols. Data quality and amplification specificity was assessed using Bio-Rad CFX Maestro software (v.4.1.24). Relative gene expression was quantified using the 2^−ΔΔCt^ method, normalizing to GAPDH as the housekeeping gene. All qPCR assays were performed in quintuplicate.

## 5. Conclusions

This study demonstrates a clear dose-dependent effect of simvastatin (SV) and fluvastatin (FV) on bone tissue dynamics within a translational organotypic bone model. At low concentrations (0.1–1 µM), both statins increased osteogenic differentiation, extracellular matrix maturation, and mineralized tissue formation. These effects were associated with the upregulation of key osteogenic markers, including RUNX2, SPP1, and COL1A2, and were supported by histological and microtomographic evidence of increased bone volume, collagen deposition and collagen maturation—without affecting chondrogenic pathways. Importantly, despite the expansion of mineralized tissue, bone mineral density remained unchanged, suggesting that statins primarily stimulate matrix production and early mineralization processes rather than altering mineral content. In contrast, higher concentrations of statins were associated with detrimental effects on cell viability and tissue development, likely due to cell cycle arrest and the activation of apoptotic pathways. Altogether, these findings underscore the therapeutic potential of statins at low concentrations and establish a foundation for their targeted application in bone repair and regenerative medicine, particularly within dosage windows that maximize anabolic outcomes while minimizing cytotoxic risks.

## Figures and Tables

**Table 1:** Primer sequences used in gene expression analysis (Bio-Rad).

Primers			
GAPDH	qGgaCED0029996	COL1A2	qGgaCED0025365
RUNX2	qGgaCID0019198	ACAN	qGgaCID0030890
SPP1	qGgaCED0023869	SOX9	qGgaCED0029640

Abbreviations: Glyceraldehyde-3-phosphate dehydrogenase (GAPDH); Runt-related transcription factor 2 (RUNX2); Secreted Phosphoprotein 1 (SPP1); Collagen type I alpha 2; Aggrecan (ACAN); SRY-box 9 (SOX9).

## Data Availability

The data that support the findings of this study are available from the corresponding author upon reasonable request.

## References

[1] Granat M.M., Eifler-Zydel J., Kolmas J. (2024). Statins-Their Role in Bone Tissue Metabolism and Local Applications with Different Carriers. Int. J. Mol. Sci..

[2] Mundy G., Garrett R., Harris S., Chan J., Chen D., Rossini G., Boyce B., Zhao M., Gutierrez G. (1999). Stimulation of bone formation in vitro and in rodents by statins. Science.

[3] Climent E., Benaiges D., Pedro-Botet J. (2021). Hydrophilic or Lipophilic Statins?. Front. Cardiovasc. Med..

[4] Salami J.A., Warraich H., Valero-Elizondo J., Spatz E.S., Desai N.R., Rana J.S., Virani S.S., Blankstein R., Khera A., Blaha M.J. (2017). National Trends in Statin Use and Expenditures in the US Adult Population From 2002 to 2013: Insights From the Medical Expenditure Panel Survey. JAMA Cardiol..

[5] Matyori A., Brown C.P., Ali A., Sherbeny F. (2023). Statins utilization trends and expenditures in the U.S. before and after the implementation of the 2013 ACC/AHA guidelines. Saudi Pharm. J..

[6] Chen P.Y., Sun J.S., Tsuang Y.H., Chen M.H., Weng P.W., Lin F.H. (2010). Simvastatin promotes osteoblast viability and differentiation via Ras/Smad/Erk/BMP-2 signaling pathway. Nutr. Res..

[7] Vukelic S., Stojadinovic O., Pastar I., Vouthounis C., Krzyzanowska A., Das S., Samuels H.H., Tomic-Canic M. (2010). Farnesyl pyrophosphate inhibits epithelialization and wound healing through the glucocorticoid receptor. J. Biol. Chem..

[8] Nes W.D. (2011). Biosynthesis of cholesterol and other sterols. Chem. Rev..

[9] Kim J.Y., Lee E.Y., Lee E.B., Lee Y.J., Yoo H.J., Choi J., Song Y.W. (2012). Atorvastatin inhibits osteoclastogenesis by decreasing the expression of RANKL in the synoviocytes of rheumatoid arthritis. Arthritis Res. Ther..

[10] Sharif P.S., Abdollahi M. (2011). A Systematic Review on the Relation between use of Statins and Osteoporosis. Int. J. Pharmacol..

[11] Zhao H., Tang Y., Zhen Y., Qi C., Chen S. (2023). The effect of statins on bone turnover biomarkers: A systematic review and meta-analysis of randomized controlled trials. Endocr. J..

[12] Bone H.G., Kiel D.P., Lindsay R.S., Lewiecki E.M., Bolognese M.A., Leary E.T., Lowe W., McClung M.R. (2007). Effects of atorvastatin on bone in postmenopausal women with dyslipidemia: A double-blind, placebo-controlled, dose-ranging trial. J. Clin. Endocrinol. Metab..

[13] Di Spirito F., Schiavo L., Pilone V., Lanza A., Sbordone L., D’Ambrosio F. (2021). Periodontal and Peri-Implant Diseases and Systemically Administered Statins: A Systematic Review. Dent. J..

[14] Pruthi G., Mahajan R., Gupta A., Patil A.N., Paramasivam V., Kaundal S. (2023). The Effects of Statins on Bone Formation Around Implants Placed in Animal Bones: A Systematic Review and Meta-Analysis. J. Maxillofac. Oral Surg..

[15] Tahamtan S., Shirban F., Bagherniya M., Johnston T.P., Sahebkar A. (2020). The effects of statins on dental and oral health: A review of preclinical and clinical studies. J. Transl. Med..

[16] Lebeaux D., Chauhan A., Rendueles O., Beloin C. (2013). From in vitro to in vivo Models of Bacterial Biofilm-Related Infections. Pathogens.

[17] Mariano L.C., Grenho L., Fernandes M.H., de Sousa Gomes P. (2023). Integrative tissue, cellular and molecular responsiveness of an innovative ex vivo model of the Staphylococcus aureus-mediated bone infection. FASEB J..

[18] Groff K., Evans S.J., Doak S.H., Pfuhler S., Corvi R., Saunders S., Stoddart G. (2021). In vitro and integrated in vivo strategies to reduce animal use in genotoxicity testing. Mutagenesis.

[19] Furtado G.S., Martin V., Araújo R., Gomes P.S., Lago A.D.N. (2024). Osteoinductive activity of photobiomodulation in an organotypic bone model. Photodiagnosis Photodyn. Ther..

[20] Araújo R., Martin V., Ferreira R., Fernandes M.H., Gomes P.S. (2022). A new ex vivo model of the bone tissue response to the hyperglycemic environment—The embryonic chicken femur organotypic culture in high glucose conditions. Bone.

[21] Cramer E.E.A., Ito K., Hofmann S. (2021). Ex vivo Bone Models and Their Potential in Preclinical Evaluation. Curr. Osteoporos. Rep..

[22] Smith E.L., Kanczler J.M., Gothard D., Roberts C.A., Wells J.A., White L.J., Qutachi Q., Sawkins M.J., Peto H., Rashidi H. (2014). Evaluation of skeletal tissue repair, part 2: Enhancement of skeletal tissue repair through dual-growth-factor-releasing hydrogels within an ex vivo chick femur defect model. Acta Biomater..

[23] Smith E.L., Kanczler J.M., Oreffo R.O.C. (2013). A new take on an old story: Chick limb organ culture for skeletal niche development and regenerative medicine evaluation. Eur. Cells Mater..

[24] Ahmed T.A., Hayslip J., Leggas M. (2013). Pharmacokinetics of high-dose simvastatin in refractory and relapsed chronic lymphocytic leukemia patients. Cancer Chemother. Pharmacol..

[25] Sabandal M.M.I., Schäfer E., Imper J., Jung S., Kleinheinz J., Sielker S. (2020). Simvastatin induces in vitro mineralization effects of primary human odontoblast-like cells. Materials.

[26] Zhang M., Bian Y.Q., Tao H.M., Yang X.F., Mu W.D. (2018). Simvastatin induces osteogenic differentiation of MSCs via Wnt/β-catenin pathway to promote fracture healing. Eur. Rev. Med. Pharmacol. Sci..

[27] Baek K.H., Lee W.Y., Oh K.W., Tae H.J., Lee J.M., Lee E.J., Han J.H., Kang M.I., Cha B.Y., Lee K.W. (2005). The effect of simvastatin on the proliferation and differentiation of human bone marrow stromal cells. J. Korean Med. Sci..

[28] Jia W., Zhao Y., Yang J., Wang W., Wang X., Ling L., Ge L. (2016). Simvastatin Promotes Dental Pulp Stem Cell-induced Coronal Pulp Regeneration in Pulpotomized Teeth. J. Endod..

[29] Gorabi A.M., Kiaie N., Pirro M., Bianconi V., Jamialahmadi T., Sahebkar A. (2021). Effects of statins on the biological features of mesenchymal stem cells and therapeutic implications. Heart Fail. Rev..

[30] Wood W.G., Igbavboa U., Muller W.E., Eckert G.P. (2013). Statins, Bcl-2, and apoptosis: Cell death or cell protection?. Mol. Neurobiol..

[31] Malik S., Sharma A.K., Bharti S., Nepal S., Bhatia J., Nag T.C., Narang R., Arya D.S. (2011). In vivo cardioprotection by pitavastatin from ischemic-reperfusion injury through suppression of IKK/NF-kappaB and upregulation of pAkt-e-NOS. J. Cardiovasc. Pharmacol..

[32] Qin W., Lu Y., Zhan C., Shen T., Dou L., Man Y., Wang S., Xiao C., Bian Y., Li J. (2012). Simvastatin suppresses apoptosis in vulnerable atherosclerotic plaques through regulating the expression of p(53), Bcl-2 and Bcl-xL. Cardiovasc. Drugs Ther..

[33] Moriishi T., Maruyama Z., Fukuyama R., Ito M., Miyazaki T., Kitaura H., Ohnishi H., Furuichi T., Kawai Y., Masuyama R. (2011). Overexpression of Bcl2 in osteoblasts inhibits osteoblast differentiation and induces osteocyte apoptosis. PLoS ONE.

[34] Lai M., Yan X., Jin Z. (2018). The response of bone cells to titanium surfaces modified by simvastatin-loaded multilayered films. J. Biomater. Sci. Polym. Ed..

[35] Li Y., Zhang Z., Zhang Z. (2018). Porous Chitosan/Nano-Hydroxyapatite Composite Scaffolds Incorporating Simvastatin-Loaded PLGA Microspheres for Bone Repair. Cells Tissues Organs.

[36] Zhang H.X., Xiao G.Y., Wang X., Dong Z.G., Ma Z.Y., Li L., Li Y.H., Pan X., Nie L. (2015). Biocompatibility and osteogenesis of calcium phosphate composite scaffolds containing simvastatin-loaded PLGA microspheres for bone tissue engineering. J. Biomed. Mater. Res. A.

[37] Zhang Y., Zhang R., Li Y., He G., Zhang D., Zhang F. (2012). Simvastatin augments the efficacy of therapeutic angiogenesis induced by bone marrow-derived mesenchymal stem cells in a murine model of hindlimb ischemia. Mol. Biol. Rep..

[38] Pirzad Jahromi G., Shabanzadeh A.P., Mokhtari Hashtjini M., Sadr S.S., Rasouli Vani J., Raouf Sarshoori J., Charish J. (2018). Bone marrow-derived mesenchymal stem cell and simvastatin treatment leads to improved functional recovery and modified c-Fos expression levels in the brain following ischemic stroke. Iran. J. Basic Med. Sci..

[39] Cai J., Yu X., Zhang B., Zhang H., Fang Y., Liu S., Liu T., Ding X. (2014). Atorvastatin improves survival of implanted stem cells in a rat model of renal ischemia-reperfusion injury. Am. J. Nephrol..

[40] Dai B., Li X., Xu J., Zhu Y., Huang L., Tong W., Yao H., Chow D.H.-K., Qin L. (2021). Synergistic effects of magnesium ions and simvastatin on attenuation of high-fat diet-induced bone loss. Bioact. Mater..

[41] Feng C., Xiao L., Yu J.C., Li D.Y., Tang T.Y., Liao W., Wang Z.R., Lu A.Q. (2020). Simvastatin promotes osteogenic differentiation of mesenchymal stem cells in rat model of osteoporosis through BMP-2/Smads signaling pathway. Eur. Rev. Med. Pharmacol. Sci..

[42] Long F. (2011). Building strong bones: Molecular regulation of the osteoblast lineage. Nat. Rev. Mol. Cell. Biol..

[43] Komori T. (2022). Whole Aspect of Runx2 Functions in Skeletal Development. Int. J. Mol. Sci..

[44] Li X., Cui Q., Kao C., Wang G.J., Balian G. (2003). Lovastatin inhibits adipogenic and stimulates osteogenic differentiation by suppressing PPARgamma2 and increasing Cbfa1/Runx2 expression in bone marrow mesenchymal cell cultures. Bone.

[45] Qiao L.J., Kang K.L., Heo J.S. (2011). Simvastatin promotes osteogenic differentiation of mouse embryonic stem cells via canonical Wnt/beta-catenin signaling. Mol. Cells.

[46] Alam S., Ueki K., Nakagawa K., Marukawa K., Hashiba Y., Yamamoto E., Sakulsak N., Iseki S. (2009). Statin-induced bone morphogenetic protein (BMP) 2 expression during bone regeneration: An immunohistochemical study. Oral Surg. Oral Med. Oral Pathol. Oral Radiol. Endodontol..

[47] Maciel-Oliveira N., Bradaschia-Correa V., Arana-Chavez V.E. (2011). Early alveolar bone regeneration in rats after topical administration of simvastatin. Oral Surg. Oral Med. Oral Pathol. Oral Radiol. Endodontol..

[48] Ruiz-Gaspa S., Nogues X., Enjuanes A., Monllau J.C., Blanch J., Carreras R., Mellibovsky L., Grinberg D., Balcells S., Díez-Perez A. (2007). Simvastatin and atorvastatin enhance gene expression of collagen type 1 and osteocalcin in primary human osteoblasts and MG-63 cultures. J. Cell. Biochem..

[49] Mansour G., Al Ashwah A., Koura A. (2014). Evaluation of simvastatin grafting around immediate dental implants in dogs. Implant. Dent..

[50] Selvaraj V., Sekaran S., Dhanasekaran A., Warrier S. (2024). Type 1 collagen: Synthesis, structure and key functions in bone mineralization. Differentiation.

[51] Dolkart O., Pritsch T., Sharfman Z.T., Somjen D., Salai M., Maman E., Steinberg E.L. (2015). The Effects of Lipophilic and Hydrophilic Statins on Bone Tissue Mineralization in Saos2 Human Bone Cell Line?In vitro Comparative Study. Pharm. Anal. Acta.

[52] Moriyama Y., Ayukawa Y., Ogino Y., Atsuta I., Koyano K. (2008). Topical application of statin affects bone healing around implants. Clin. Oral Implant. Res..

[53] Moriyama Y., Ayukawa Y., Ogino Y., Atsuta I., Todo M., Takao Y., Koyano K. (2010). Local application of fluvastatin improves peri-implant bone quantity and mechanical properties: A rodent study. Acta Biomater..

[54] Gruber H.E., Ingram J., Hanley E.N. (2002). An improved staining method for intervertebral disc tissue. Biotech. Histochem..

